# Evolutionary History and Diversification of M35 Metalloproteases in Dothideomycetes: A Phylogenomic Overview and Case Study in *Corynespora cassiicola*

**DOI:** 10.1007/s00284-026-04772-x

**Published:** 2026-02-21

**Authors:** Vinicius D. Rocha, Thaís C. S. Dal’Sasso, Maximiller D. B. L. Costa, Luiz Orlando de Oliveira

**Affiliations:** 1 Departamento de Bioquímica e Biologia Molecular, Universidade Federal de Viçosa, Viçosa, Brazil; 2 Environmental Genomics Group, Christian-Albrechts University of Kiel, and The Max Planck Institute for Evolutionary Biology, Plön, Germany

## Abstract

Deuterolysin metalloproteases (M35s), a family of zinc-dependent proteolytic enzymes, function as key virulence factors in bacteria and fungi. In plant-pathogenic fungi, these proteins induce host cell death and suppress plant chitinases, thereby protecting fungal cell walls from degradation and preventing the release of chitin oligomers that trigger plant immune responses. Here, we investigated the evolutionary history of the M35 gene family across Dothideomycetes fungi using genomic data from 79 Dothideomycetes species and 61 *Corynespora cassiicola* isolates. Predicted M35 proteins were classified as putative effectors or putative non-effectors based on bioinformatic criteria, with putative effectors defined as secreted proteins capable of manipulating host immunity. Across Dothideomycetes, we identified 146 M35 genes, of which 107 were putative effectors and 39 putative non-effectors. Family size varied widely (0–7 genes per genome), with the largest repertoires observed in the plant-pathogenic species *Botryosphaeria dothidea* and *Diplodia seriata*. In *C. cassiicola*, most isolates harbored three M35 genes per genome. Phylogenetic analysis revealed four distinct sub-clades within the *C. cassiicola* M35 family. Sub-clades Cc_M35_1.1 and Cc_M35_2.1 showed evidence of gene duplication followed by retention across most isolates, whereas Cc_M35_1.2 and Cc_M35_2.2 exhibited patterns consistent with gene loss. Notably, putative effector M35s in sub-clade Cc_M35_2.2 were exclusively recovered from isolates associated with cotton, suggesting a specialized role in *C. cassiicola*–cotton interactions. During soybean infection, all three M35 genes in *C. cassiicola* were expressed, but CC_29_g9669 (Cc_M35_1.1) displayed the highest relative expression, peaking at 8 days post-inoculation.

**Supplementary Information:**

The online version contains supplementary material available at 10.1007/s00284-026-04772-x.

## Introduction

Deuterolysin metalloproteases (M35s) are zinc-dependent enzymes classified in the M35 family according to the MEROPS peptidase database [1]. While their precise catalytic mechanisms remain unclear, M35s share a conserved active site comprising two histidine residues and one aspartic acid coordinated to a zinc ion [1]. Structurally, these enzymes are defined by the conserved Peptidase_M35 domain (PF02102; IPR001384), which contains two signature motifs: HEXXH and GTXDXXYG [1]. The HEXXH motif includes two zinc-binding histidines and a catalytic glutamate, whereas the GTXDXXYG motif provides a third zinc-binding aspartic acid residue [1, 2].

M35 metalloproteases play diverse roles in pathogenicity in bacteria and fungi [3–6]. In the fish-pathogenic bacterium *Aeromonas salmonicida* subsp. *achromogenes*, the secreted M35 protein AsaP1 exhibits strong cytotoxicity toward fish cells, although its molecular mechanism of action remains unresolved [3]. Deletion of the AsaP1-encoding gene reduces virulence, underscoring its role as a key virulence factor [3]. In fungi, M35s have similarly been implicated in host–pathogen interactions. In the plant-pathogenic fungus *Verticillium dahliae*, the M35-encoding genes *VdM35-1* and *VdASPF2* trigger host immune responses, including hypersensitive cell death; their deletion reduces virulence, spore production, and mycelial growth [5]. Likewise, in the entomopathogenic fungus *Metarhizium robertsii*, only one of seven M35 genes (*MrM35-4*) is essential for virulence, inducing host cell apoptosis and suppressing insect immunity by cleaving prophenoloxidase enzymes [6].

Accumulating evidence highlights M35s as important virulence factors in plant–fungus interactions, particularly through their ability to suppress plant chitinases [7, 8]. Plant chitinases degrade fungal cell walls by hydrolyzing chitin and generate chitin oligomers that act as potent immune elicitors [9]. These oligomers are recognized by plant pattern recognition receptors (PRRs), triggering chitin-induced immunity that involves MAPK signaling cascades and the accumulation of reactive oxygen species [10, 11]. To counteract this defense, pathogenic fungi deploy M35 proteins that inhibit chitinase activity, thereby protecting fungal cell wall integrity and preventing activation of host immune responses [7, 8]. For example, *Fusarium oxysporum* f. sp. *cubense* secretes the M35 effector FocM35_1, which inhibits banana chitinases in vitro and specifically interacts with the MaChiA chitinase [7]. FocM35_1 is secreted into the apoplast and induces cell death when transiently expressed in *Nicotiana benthamiana* leaves [7]. Similarly, RcMEP2 from *Rhizoctonia cerealis* suppresses host chitinase expression and induces cell death in wheat leaves [8].

The evolutionary history of the M35 gene family reflects its functional importance in pathogenic fungi. Previous studies have shown that M35 genes have undergone gene duplication and positive selection in vertebrate-pathogenic fungi such as *Coccidioides posadasii* and *Coccidioides immitis* [12]. A broader survey of 50 Ascomycota species revealed marked variation in M35 gene copy number, ranging from zero to seven genes per species [4]. Within the order Onygenales, which includes several vertebrate pathogens, the M35 family has experienced dynamic cycles of gene duplication and gene loss [4]. Despite these insights, the biological roles of M35s in vertebrate-pathogenic fungi remain incompletely understood.

Elucidating the evolutionary history of the M35 gene family can clarify how gene duplication, loss, and diversification have shaped their present-day distribution and functional specialization. Our recent phylogenomic reconstruction of 79 Dothideomycetes species provides an ideal framework for such analyses [13]. Dothideomycetes is one of the largest and most diverse classes within Ascomycota, comprising over 19,000 species distributed across 23 orders and encompassing a wide range of lifestyles, including pathogenic, endophytic, saprobic, mycorrhizal, and lichenized forms [14]. The class likely originated from a saprophytic ancestor and has undergone multiple independent transitions to plant pathogenicity [14]. Three orders in particular—Botryosphaeriales, Capnodiales, and Pleosporales—contain numerous economically important plant pathogens [13, 15]. The order Botryosphaeriales comprises polyphagous plant-pathogenic fungi such as *Botryosphaeria dothidea* and *Diplodia seriata*, whereas Capnodiales includes damaging pathogens such as the wheat pathogen *Zymoseptoria tritici* and the tomato leaf mold fungus *Cladosporium fulvum*. Pleosporales is the largest of these orders, accounting for approximately 25% of all Dothideomycetes species [16].

Within Pleosporales, *Corynespora cassiicola* (Corynesporascaceae) stands out as a versatile pathogen capable of infecting more than 400 plant species, including soybean, cotton, and tomato [17]. It causes target spot disease, characterized by necrotic leaf lesions with yellowish-green halos and severe defoliation, and is also responsible for Corynespora leaf fall in rubber trees (*Hevea brasiliensis*) in Asia and Africa [17]. Notably, infections by *C. cassiicola* have also been reported in nematodes and humans [17]. Our previous work generated genomic data for multiple *C. cassiicola* isolates [18, 19], and we recently completed the genome sequencing of *Corynespora smithii* isolate CBS 139,925 (Dal’Sasso et al., unpublished), a closely related species to *C. cassiicola* [20]. Together, these genomic resources offer a valuable opportunity to investigate the evolution of fungal gene families, including M35 metalloproteases.

In this study, we examined the evolutionary history of the M35 gene family using two complementary approaches: a broad phylogenomic analysis across Dothideomycetes and a focused, population-level analysis within *C. cassiicola*. Predicted M35 proteins were classified as putative effector or non-effector M35s, adopting a broad definition of effectors as secreted molecules that manipulate host cell structures and immune responses to facilitate infection [21, 22]. Using genomic data from 79 Dothideomycetes species, we analyzed the distribution of M35 genes, family size variation, and phylogenetic relationships. We further investigated M35 evolution in *C. cassiicola* using genomes from 61 isolates and one isolate of *C. smithii*, assessing patterns of gene retention, gene loss, and interspecific relationships. Finally, we examined the expression profiles of *C. cassiicola* M35 genes during soybean infection using RT-qPCR. Together, our analyses provide new insights into the evolutionary mechanisms driving the diversification of M35 metalloproteases in Dothideomycetes fungi.

## Materials and Methods

### Data Compilation and Gene Prediction

To investigate the evolutionary history of the M35 gene family in Dothideomycetes, protein sequences from 79 species (one per genus) were retrieved from the MycoCosm portal [23] (Supplementary Table S1). This dataset has been used in previous phylogenetic studies [13, 19]. Protein sequences from *Aspergillus nidulans* and *Aspergillus fumigatus* (Eurotiomycetes) were included as outgroups.

For single-species analyses, genome data from 61 *C. cassiicola* isolates were compiled. Genome sequences from 14 isolates were obtained from GenBank, while predicted coding sequences (CDSs) and protein sequences from 44 isolates were taken from a previous study [18] (Supplementary Table S2). CDSs and protein sequences from three additional *C. cassiicola* isolates (CC_08, CC_10, and CC_28) were also included (Dal’Sasso et al., unpublished data). In addition, CDSs and protein sequences from *Corynespora smithii* isolate CBS 139,925 were incorporated (Dal’Sasso et al., unpublished data).

Gene prediction for genomes retrieved from GenBank was performed using Augustus v3.2.2 [24], following a previously described approach [18], with the *C. cassiicola* isolate CCP used as the pre-trained species model for the “--species” parameter.

### Protein Annotation and Secretome Prediction

Protein annotation and secretome prediction were performed using a customized pipeline described previously [18]. Predicted proteins were annotated using PfamScan with the Pfam database v33.0 [25] and InterProScan v5.30.69 [26]. InterProScan analyses included the following databases: SMART-7.1, SUPERFAMILY-1.75, ProDom-2006.1, CDD-3.16, TIGRFAM-15.0, Pfam v31.0, Coils-2.2.1, and Gene3D-4.2.0.

Putative secreted proteins were identified based on the presence of a signal peptide predicted by SignalP v4.1 [27] and the absence of transmembrane domains as determined by TMHMM v2.0c [28]. Subcellular localization of predicted secreted proteins was assessed using TargetP v1.1b [29].

### Identification of M35 Proteins

Members of the M35 gene family were identified using local BLASTp searches (e-value cutoff 1e-4) against the MEROPS database v12.4 [1], with fungal proteomes as queries. In parallel, HMMER v3.3.2 (http://hmmer.org/*)* was used to detect M35 homologs (e-value < 0.001) based on profile Hidden Markov Models for the Peptidase_M35 domain (PF02102) obtained from Pfam v33.0 [25]. A protein was classified as an M35 member if it was annotated as M35 in MEROPS (MER0001394) and contained the Peptidase_M35 domain identified by at least two of the following tools: PfamScan, InterProScan, and HMMER.

Moreover, each predicted M35 protein was classified as either a putative effector or a putative non-effector. To classify an M35 protein as a putative effector, four criteria were applied: (1) presence of a signal peptide predicted by SignalP, (2) absence of transmembrane domains as determined by TMHMM, (3) localization within the secretory pathway predicted by TargetP, and (4) protein localization predicted as ‘extracellular’ by DeepLoc2 [30]. An M35 protein was considered a putative effector only if it met all four criteria. Proteins failing to meet these criteria were categorized as putative non-effector M35 proteins.

### Datasets and Alignments

Five datasets of M35 sequences were constructed. Dataset A included 146 full-length protein sequences (1536 amino acids) from 65 Dothideomycetes species and two *Aspergillus* spp. Dataset B comprised 63 full-length protein sequences (830 amino acids) from 31 species within Pleosporales. Three datasets focused on *Corynespora*: dataset C contained 165 CDSs (2602 bp) from 61 *C. cassiicola* isolates and two CDSs from *C. smithii*; dataset D consisted of 165 CDSs (2594 bp) from the 61 *C. cassiicola* isolates; and dataset E included 165 protein sequences (675 amino acids) from the same isolates. Sequence alignments for all datasets were generated using the L-INS-i algorithm in MAFFT v7.453 [31] with default parameters.

### Phylogenetic Reconstructions

Maximum likelihood (ML) phylogenetic analyses were conducted to infer evolutionary relationships among M35 genes in Dothideomycetes, Pleosporales, and *C. cassiicola*. Dataset A and dataset B were used to reconstruct M35 phylogenies across Dothideomycetes and Pleosporales, respectively, while dataset C was used to infer relationships within *C. cassiicola* and *C. smithii*.

The best-fit evolutionary models were selected using ModelFinder 2 [32] implemented in IQ-TREE v1.6.12 [33], based on the Bayesian Information Criterion. The selected models were LG + R6 (dataset A), LG + I+G4 (dataset B), and HKY + F+G4 (dataset C). ML phylogenies were inferred in IQ-TREE v1.6.12 [33] using ten independent runs and 1000 ultrafast bootstrap replicates, with a minimum bootstrap convergence correlation coefficient of 0.99. Consensus trees were generated and visualized using FigTree v1.2.4 (http://tree.bio.ed.ac.uk/software/figtree/*).*

### Gene Genealogies and Nucleotide Diversity Analyses

Gene genealogies of M35 CDSs from *C. cassiicola* were inferred using Network v5.0.03 (Fluxus Technology Ltd) with the median-joining method [34] and default parameters. Indels were not considered as a source of information in these analyses. Dataset D was used to infer overall genealogical relationships and was subsequently subdivided into haplogroup-specific sub-datasets based on the initial analysis. Genealogies were reconstructed separately for each haplogroup. For each haplogroup, DnaSP v6 [35] was used to estimate nucleotide diversity parameters, including the number of segregating sites (S), number of haplotypes (H), haplotype diversity (Hd), nucleotide diversity (π), and average number of nucleotide differences (K).

### Identification of Conserved Motifs

To identify conserved motifs in the M35 proteins of *C. cassiicola*, we utilized dataset E. Motif prediction was performed using GLAM2 and GLAM2SCAN tools available in the MEME suite v5.0.5 [36] with default parameters. First, GLAM2 analyzed the set of M35 proteins and generated motifs. Subsequently, GLAM2SCAN searched for matches to the motifs identified by GLAM2. For each match, a score was reported to assess significance. Finally, motifs with high scores were selected as the conserved motifs of interest.

### Expression Profiling of M35 Genes in *C. Cassiicola* during Soybean Interaction

RT-qPCR was used to assess the expression of M35 genes in *C. cassiicola* during interaction with the susceptible soybean cultivar BR/MG Conquista, following established protocols [18]. The *C. cassiicola* isolate CC_29, originally collected from soybean leaves in Brazil [37], was used for all experiments.

Soybean plants were grown in a greenhouse and inoculated at the V3 stage using a conidial suspension (3.5 × 10⁴ conidia/mL) containing 0.01% Tween 20. Four droplets (20 µL) were applied to the abaxial surface of each leaflet. Leaf disks (1.4 cm²) were collected from inoculation spots at 0, 2, 4, 6, and 8 days post-inoculation (dpi). Four biological replicates (individual plants) were used for each time point. As a control, plants were inoculated with distilled autoclaved water containing 0.01% Tween 20.

Infected leaf disks were macerated in liquid nitrogen, and total RNA was extracted using TRIzol Reagent (Life Technologies). RNA concentrations were quantified using a NanoDrop 2000 (Thermo Fisher), and integrity was verified on a 1.5% (w/v) agarose gel. Genomic DNA was removed with DNase I Amp Grade (Invitrogen). cDNA synthesis was performed using the M-MLV Reverse Transcriptase kit (Invitrogen) with 4 µg of RNA per reaction.

Primers for M35 genes were designed using Primer3Plus [https://www.primer3plus.com/], and their specificity was confirmed via melt curve analysis. Primer sequences are provided in Supplementary Table S3. RT-qPCR assays were performed on a 7500 Real-Time PCR System (Applied Biosystems) using PowerUp SYBR Green Master Mix, primers (1.5 µM), and 1 µL of 2-fold diluted cDNA. Amplification conditions were 50 °C for 2 min, 95 °C for 10 min, followed by 40 cycles of 94 °C for 15 s and 60 °C for 1 min.

Gene expression was normalized to the endogenous β-tubulin gene as previously described [18], and relative expression levels were calculated using the 2^⁻∆∆Ct^ method [38]. Statistical differences were assessed by one-way ANOVA followed by Tukey’s test in R v4.2.2 (https://www.r-project.org/*).* Heatmaps were generated using ggplot2 in R.

## Results

### Genomic Landscape of the M35 Gene Family in Dothideomycetes

Analysis of complete genomes from 79 Dothideomycetes species revealed a total of 139 predicted M35 genes, whereas the two *Aspergillus* spp. genomes contained seven genes in total (Supplementary Table S1). Each predicted M35 gene possessed a single Peptidase_M35 domain (PF02102). In the Dothideomycetes, all 139 M35 genes encoded proteins containing three zinc-binding residues (304 H, 308 H, 319D) and a catalytic residue (304E) (Supplementary Table S4). These residues were also present in the majority of M35s (5 out of 7) identified in *Aspergillus* spp.; however, two M35s in *Aspergillus* spp. lacked the zinc-binding residue 319D.

The size of the M35 gene family varied across Dothideomycetes, with up to seven copies per genome (Supplementary Figure S1 and Table S1). The largest M35 gene families were observed in two plant-pathogenic species within the order Botryosphaeriales: *B. dothidea* (seven members) and *D. seriata* (six members). In contrast, the M35 gene family was absent from the genomes of 14 species across six orders, including Capnodiales (six species), Botryosphaeriales (one), Dothidiales (one), Myriangiales (two), Mytilinidiales (one), and Pleosporales (three). Among the Pleosporales species, the M35 gene family comprised three members in 12 species, including *C. cassiicola* (isolate CCP).

### Predicted Putative Effector and Putative non-effector M35s

In silico analyses identified a total of 107 M35s as putative effectors (Supplementary Figure S1 and Table S1). The number of putative effector M35s varied among the studied genomes, with a maximum of four in two species, except for *D. seriata* (order Botryosphaeriales), which had five. Within Pleosporales, *C. cassiicola* (isolate CCP) and eight additional species contained three putative effector M35s.

Putative non-effector M35s were relatively uncommon, with a total of 39 identified (Supplementary Figure S1 and Table S1). Seven species from five different orders harbored exclusively putative non-effector M35s: one species each from Aulographales and Botryosphaeriales, two from Histeriales, one from Micothyriales, and two from Pleosporales. Interestingly, many genes encoding putative non-effector M35s exhibited large indels at the 5’-end, resulting in the absence of a signal peptide for extracellular secretion.

### Phylogenetic Analyses of M35 Proteins

The maximum likelihood (ML) phylogenetic tree reconstructed relationships among 139 M35 proteins (dataset A) from 65 Dothideomycetes species, along with seven M35 proteins from *Aspergillus* spp. (Supplementary Figure S2). The tree revealed three major clades; however, these clades received low bootstrap support (< 70). We attributed the low nodal support to high sequence divergence among M35 proteins, which introduced a high noise-to-signal ratio in dataset A. To mitigate this, we focused subsequent phylogenetic analysis on Pleosporales (dataset B), the most species-rich order in our Dothideomycetes sampling and the order that includes the genus *Corynespora*.

**Fig. 1:**
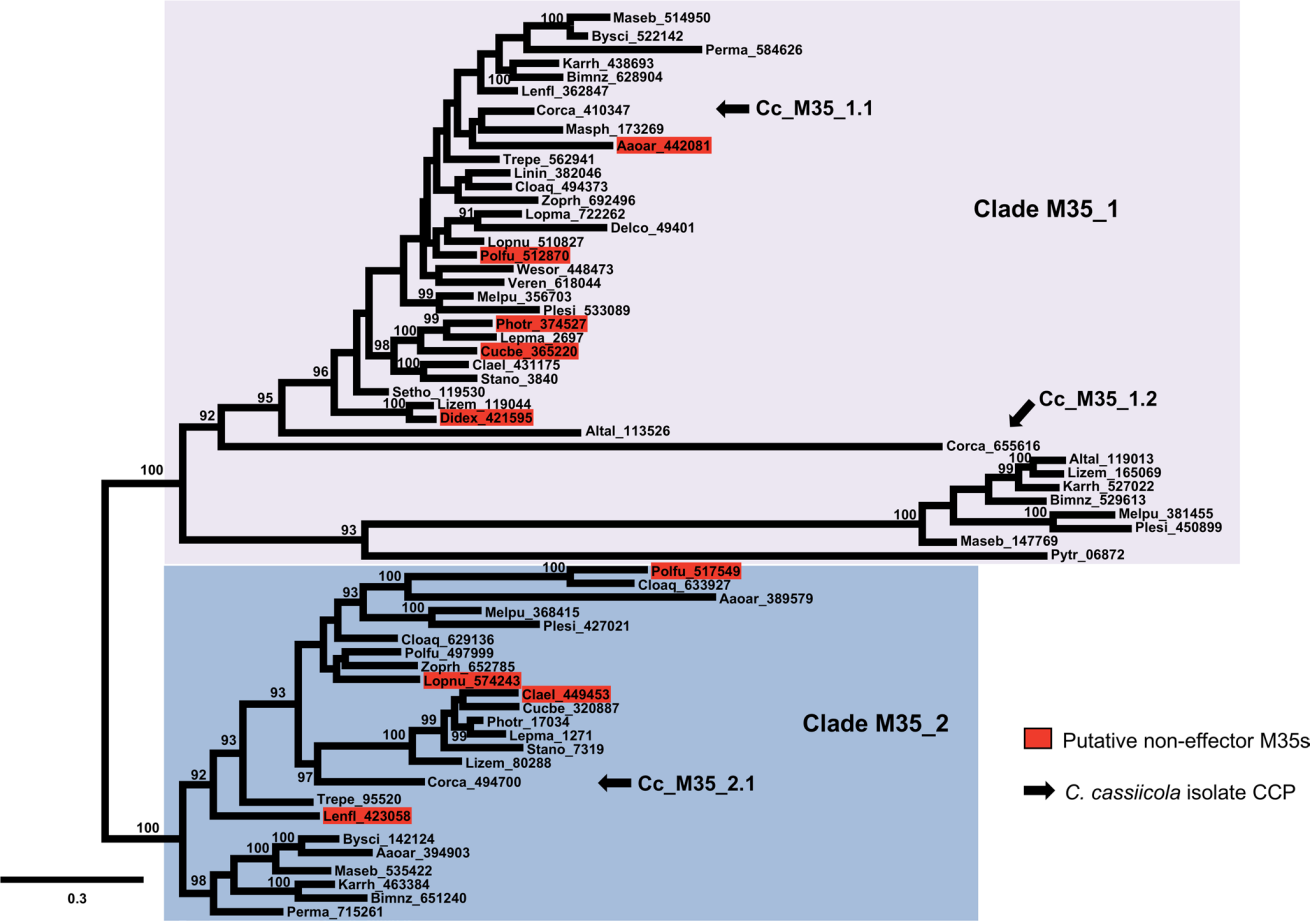
Maximum likelihood tree showing the two clades (M35_1 and M35_2) of the Deuterolysin Metalloprotease (M35) gene family across the Pleosporales. The unroot consensus tree was based on 63 protein sequences of members of the M35 gene family from 31 species of Pleosporales. Proteins contained 830 amino acids. Nodal support values are given as bootstrap values above the branches when > 80. Scale bar corresponds to the expected number of substitutions per site. Arrows indicate the three predicted M35 genes (Cc_M35_1.1, Cc_M35_1.2, and Cc_M35_2.1) identified in the genome of *Corynespora cassiicola* isolate CCP. Proteins were predicted to be putative effector M35s; red highlights indicate putative non-effector M35s

Using 63 M35 proteins from 31 Pleosporales species, we reconstructed an ML phylogenetic tree with well-supported nodes (bootstrap values > 90 for major nodes; Fig. 1). This tree comprised two major clades, designated M35_1 and M35_2. Clade M35_1 was the most populous, containing 39 members (34 putative effector M35s and 5 putative non-effector M35s). Members of clade M35_1 were present in 30 of the 31 Pleosporales species (Fig. 1); among these, 23 species contained a single member in clade M35_1, while the remaining eight species had two. In *C. cassiicola* isolate CCP, the clade M35_1 members were named Cc_M35_1.1 and Cc_M35_1.2. In contrast, clade M35_2 consisted of 24 members (20 putative effector M35s and 4 putative non-effector M35s) from 22 Pleosporales species (Fig. 1). The third M35 gene in *C. cassiicola* (isolate CCP), named Cc_M35_2.1, was placed within clade M35_2.

### The M35 Gene Family in *Corynespora*

Our analysis recovered 165 M35 genes across 61 *C. cassiicola* isolates (Supplementary Table S2). The number of M35 genes per genome ranged from 1 to 4, with most isolates (42 out of 61) harboring three. Isolate CC_28 was exceptional, containing four M35 genes. Of the 165 M35 genes, 119 were classified as putative effectors and 46 as putative non-effectors. The genome of the closely related species *C. smithii* contained two M35 genes: one putative effector and one putative non-effector.

**Fig. 2:**
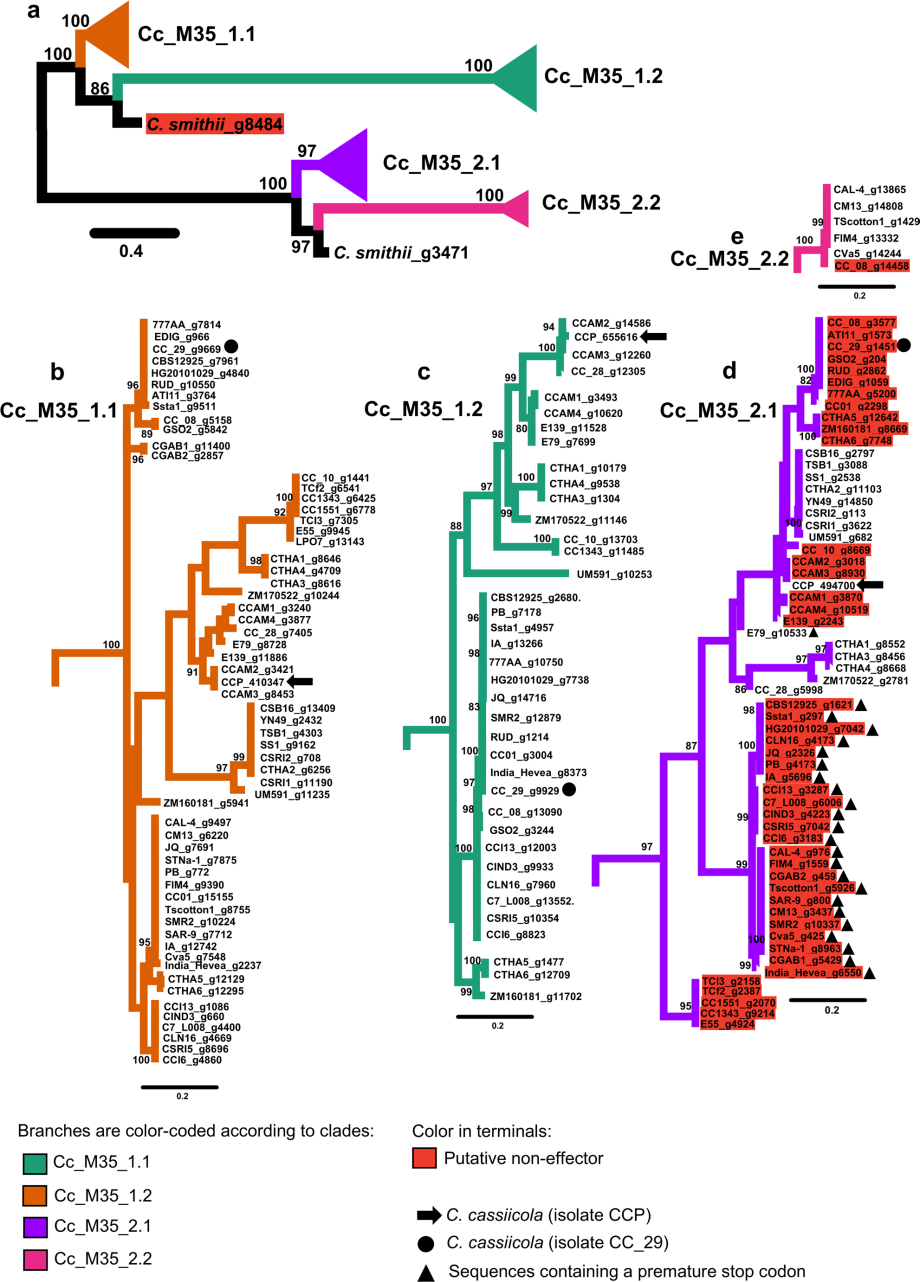
Phylograms of the maximum likelihood tree of Deuterolysin Metalloprotease (M35) gene family across the 61 isolates of *Corynespora cassiicola* and one single isolate of *Corynespora smithii* CBS 139,925. The analysis was based on 165 coding DNA sequences (each of which contained 2602 base pairs long) of members of the M35 gene family from 62 isolates. **a** Overview of the full unrooted consensus tree, with four major sub-clades collapsed into triangles. **b-e** Expanded views show the composition of each major sub-clade. **b** Cc_M35_1.1 (orange). **c** Cc_M35_1.2 (green). **d** Cc_M35_2.1 (purple). **e** Cc_M35_2.2 (pink). Nodal support values are given as bootstrap values above the branches, when > 80. Branch lengths are drawn to scale. Scale bar corresponds to the expected number of substitutions per site. Red terminals indicate putative non-effectors M35. Placements of predicted M35 family members of isolates CCP and CC_29 are as indicated

An ML phylogenetic tree was constructed using CDS sequences of the 165 M35 genes (dataset C) from *C. cassiicola*, along with two genes from *C. smithii* (total of 167 sequences; Fig. 2a). The tree revealed two major clades, each with 100% bootstrap support. Each clade included one M35 gene from *C. smithii*, which formed a sister relationship to the *C. cassiicola* M35 genes. Within each clade, *C. cassiicola* M35 genes formed distinct sub-clades, designated Cc_M35_1.1, Cc_M35_1.2, Cc_M35_2.1, and Cc_M35_2.2 (Fig. 2a).

Sub-clade Cc_M35_1.1 was the most gene-rich, containing one gene from each of the 61 *C. cassiicola* isolates (Fig. 2b). Its sister sub-clade, Cc_M35_1.2, was smaller, with 38 members (Fig. 2c). All members of Cc_M35_1.1 and Cc_M35_1.2 encoded putative effector M35s.

In contrast, sub-clade Cc_M35_2.1 contained 60 members, including 15 putative effector M35s and 45 putative non-effector M35s (Fig. 2d). Several second-order sub-clades within Cc_M35_2.1 consisted exclusively of putative non-effectors; members of these sub-clades were sampled from a wide variety of hosts and countries. One such second-order sub-clade comprised 23 members with a premature stop codon located 189 bp upstream of the initiation codon. These 23 genes originated from isolates collected from diverse hosts (cotton, cucumber, soybean, and rubber tree) across the Americas (USA and Brazil), Africa (Côte d’Ivoire and Gabon), and Asia (China, India, Malaysia, and Sri Lanka) [41].

The smallest sub-clade, Cc_M35_2.2, consisted of only six members (Fig. 2e). Five were putative effector M35s from isolates collected from cotton in the USA, while the sixth (a putative non-effector) was from isolate CC_28, collected from coleus (*Plectranthus barbatus*) in Brazil.

### Haplogroup Diversity of M35 Genes in *Corynespora*

**Fig. 3:**
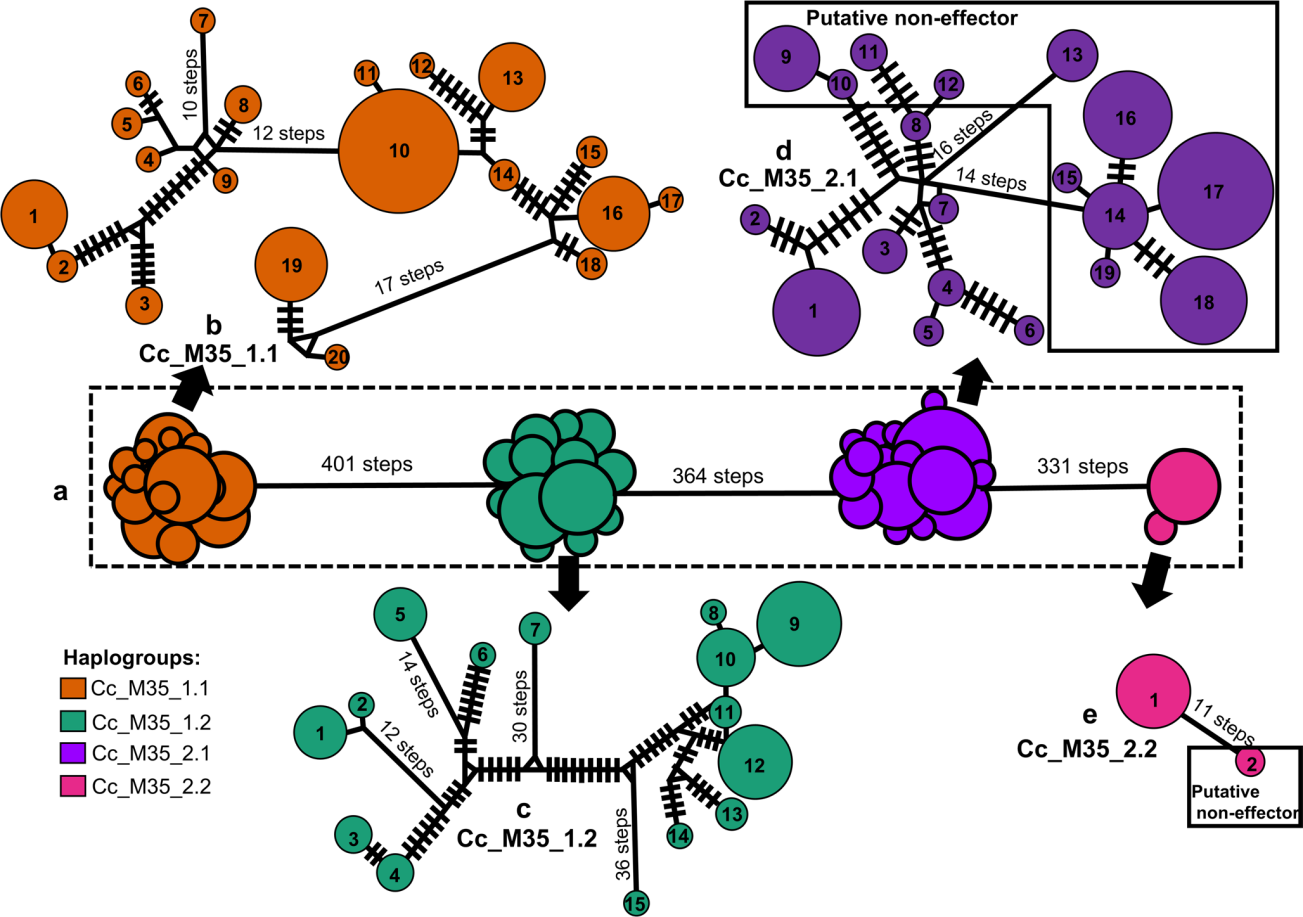
Genealogical relationships of the four haplogroups of the Deuterolysin Metalloprotease (M35)-encoding genes (CDS) of *Corynespora cassiicola*. **a** The central box depicts the full median-joining network. **b-e** Expanded views show the composition of each haplogroup. The four haplogroups are color-coded according to the predicted M35 encoding-genes: **b** Cc_M35_1.1 (orange). **c** Cc_M35_1.2 (green). **d** Cc_M35_2.1 (purple). **e** Cc_M35_2.2 (pink). A circle represents a given haplotype (coded with a number); circle size is proportional to the relative frequencies. Numbers of mutational steps are indicated with bars when more than one (unless indicated otherwise)

Among the 165 M35-encoding genes from 61 *C. cassiicola* isolates (dataset D), 56 haplotypes were identified, forming four distinct haplogroups (Fig. 3a). Haplogroup distribution closely mirrored the sub-clades from the previous phylogenetic analysis (Fig. 2), with each haplogroup corresponding to one sub-clade: Cc_M35_1.1, Cc_M35_1.2, Cc_M35_2.1, and Cc_M35_2.2.

In the network representation, haplogroups Cc_M35_1.1 and Cc_M35_2.2 occupied opposite ends, whereas Cc_M35_1.2 and Cc_M35_2.1 were positioned centrally (Fig. 3a). The largest mutational distance (401 steps) occurred between tip haplogroup Cc_M35_1.1 and central haplogroup Cc_M35_1.2, while the smallest (331 steps) was between central Cc_M35_2.1 and tip Cc_M35_2.2.

Partial networks revealed internal structure within each haplogroup (Fig. 3b–e). Tip haplogroup Cc_M35_1.1 was the most diverse (20 haplotypes; Fig. 3b), whereas Cc_M35_2.2 had only two (Fig. 3e). Haplotypes in Cc_M35_1.2 showed high divergence (up to 36 mutational steps; Fig. 3c). Haplotypes encoding putative effector M35s predominated in Cc_M35_1.1 and Cc_M35_1.2, whereas those encoding putative non-effectors were mainly in Cc_M35_2.1 (haplotypes 9–19) and Cc_M35_2.2 (haplotype 2). One haplotype (haplotype 3) in Cc_M35_2.1 was shared by both putative effector and non-effector M35 genes (Supplementary Table S2). Haplotype placement in the overall network showed no clear association with host species or geographic origin of the isolates (data not shown).

Molecular diversity analyses revealed distinct polymorphism levels among haplogroups (Table 1). Haplogroup Cc_M35_1.2 exhibited the highest nucleotide diversity (π = 0.03048) and average number of nucleotide differences (K ≈ 16), whereas Cc_M35_2.2 showed the lowest (π = 0.00349; K ≈ 3).

**Table 1:** Segregating sites (S), number of haplotypes (H), haplotype diversity (Hd), nucleotide diversity (π), and average number of nucleotide differences (K) for four deuterolysin metalloprotease (M35) haplogroups in *Corynespora Cassiicola*

Haplogroups	S	H	Hd	π	K
Cc_M35_1.1	73	20	0.921	0.01528	16.18
Cc_M35_1.2	134	15	0.925	0.03048	26.64
Cc_M35_2.1	68	19	0.923	0.01415	14.86
Cc_M35_2.2	11	2	0.333	0.00349	3.66

### Structural Features of M35 Proteins in *Corynespora*

**Fig. 4:**
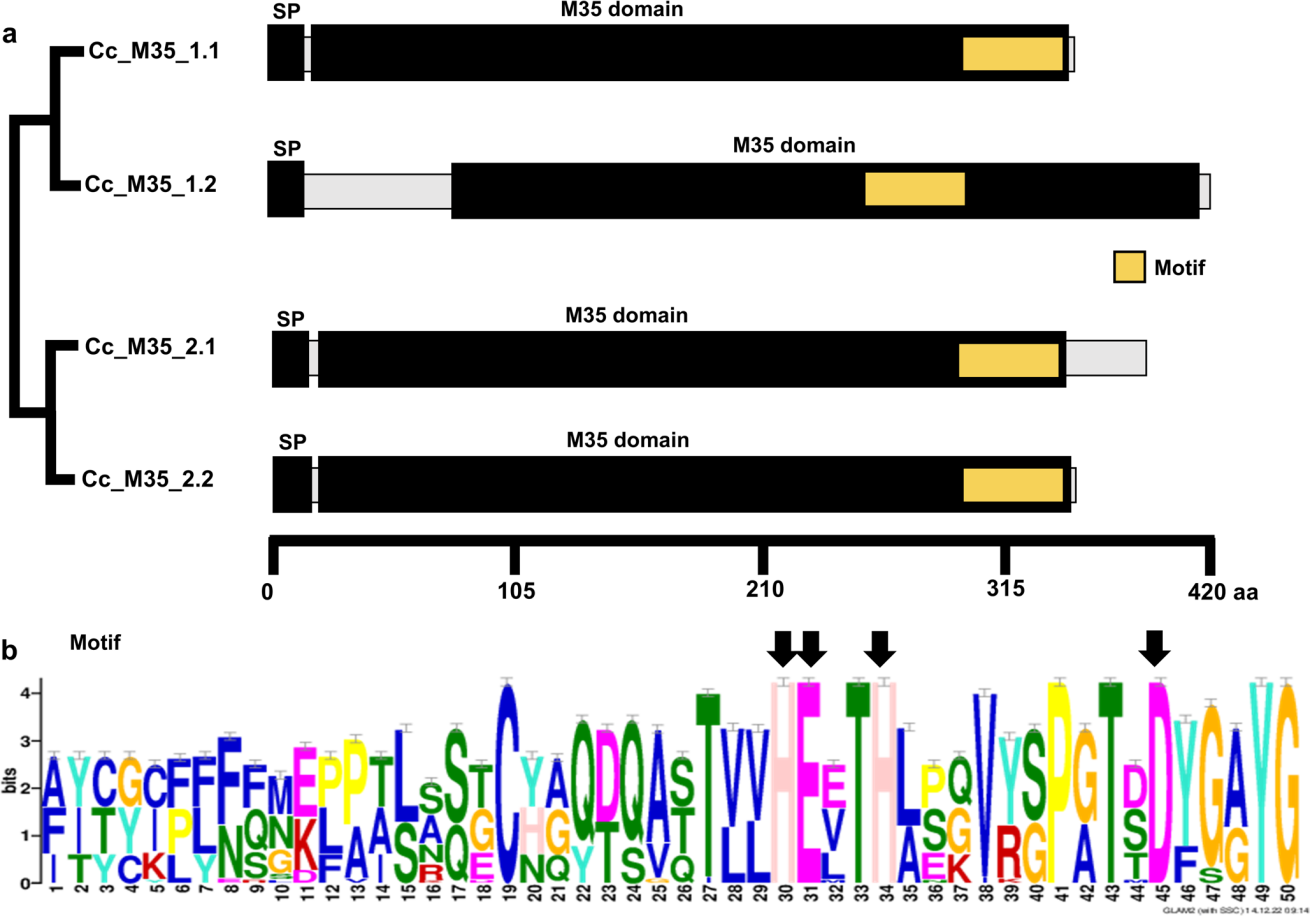
Structural overview of the Deuterolysin Metalloprotease (M35) family members of *Corynespora cassiicola*. **a** Schematic diagram depicts the average length of the putative effector M35s, the signal peptide (SP), and the predicted locations for the Peptidase_M35 domain (PF01095) and its structural motif. Protein lengths were drawn to scale. Scale bar indicates the number of amino acid residues. Schematic view of the phylogenetic tree according to Fig. 2. **b** Consensus pattern for predicted M35 motif. Arrows indicate amino acid residues involved in the active site

Figure 4a illustrates the protein structure of putative effector M35s in *C. cassiicola*. On average, full-length putative effector M35s comprised 380 amino acid residues, ranging from 349 (Cc_M35_2.2) to 420 (Cc_M35_1.2). SignalP predicted signal peptides of 18 (Cc_M35_1.1, Cc_M35_1.2, Cc_M35_2.1) to 21 (Cc_M35_2.2) amino acid residues.

HMMER analyses identified a single Peptidase_M35 domain in putative effector M35s, varying in size from 316 (Cc_M35_2.1) to 344 (Cc_M35_1.1) amino acid residues. A conserved motif encompassing 50 amino acid residues was identified toward the C-terminal region of the Peptidase_M35 domain in both putative effectors and non-effectors (Fig. 4b). This motif included three zinc-binding residues (30 H, 34 H, 45D) and a catalytic residue (31E).

### Expression Patterns of M35 Genes in *Corynespora*

**Fig. 5:**
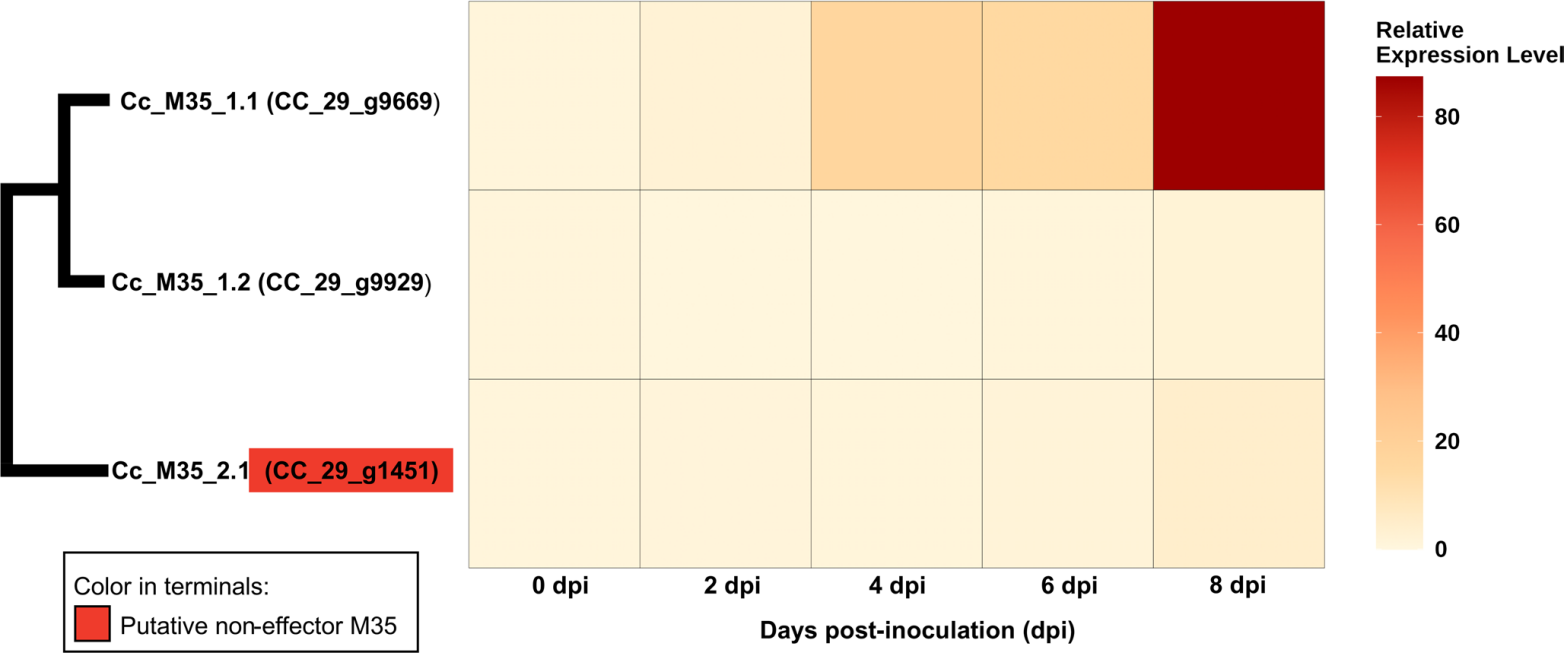
Heatmap showing the relative expression level of the three Deuterolysin Metalloprotease (M35) genes of *Corynespora cassiicola* after spore inoculation on soybean leaves. The relative expression level was calculated using the 2^−∆∆Ct^ method. The constitutive β-tubulin gene of *C. cassiicola* was used as an endogenous control. The x-axis shows the five-time points of the expression analyses: 0, 2, 4, 6, and 8 days post-inoculation (dpi). The y-axis shows the three M35 genes: Cc_M35_1.1 (CC_29_g9669). Cc_M35_1.2 (CC_29_9929). Cc_M35_2.1 (CC_29_g1451). Schematic view of the phylogenetic tree according to Fig. 2. Color in terminal: red highlights indicate a putative non-effector M35. Color intensity corresponds to relative expression level. Heatmap was generated according to the results shown in Supplementary Figure S3

RT-qPCR assays assessed temporal expression patterns of the three M35 genes in *C. cassiicola* isolate CC_29 during infection of susceptible soybean plants. All three genes were expressed during infection, but expression levels differed markedly (Fig. 5 and Supplementary Figure S3). Gene CC_29_g9669 (Cc_M35_1.1 haplogroup/sub-clade) exhibited the highest relative expression, increasing progressively from 2 dpi and peaking at 8 dpi. In contrast, CC_29_g9929 (Cc_M35_1.2) and CC_29_g1451 (Cc_M35_2.1) showed low relative expression throughout the infection process (0–8 dpi), with only a slight increase at 8 dpi.

## Discussion

### The M35 Gene Family: Insights from Size Variations and lineage-specific Expansion

Gene family size is subject to fluctuations driven by gene duplications and losses. The number of M35 family members varied substantially among Dothideomycetes species (Supplementary Figure S1). For instance, *Septoria musiva* contained a single M35 gene, whereas *B. dothidea* harbored seven. Such variation likely results from gene duplication and loss events, which have been identified as major drivers of M35 gene family evolution in certain fungal lineages, including the order Onygenales within Eurotiomycetes [4].

Lineage-specific expansion of the M35 gene family was particularly evident in the order Botryosphaeriales, where two plant-pathogenic species (*B. dothidea* and *D. seriata*) exhibited the largest repertoires. This redundancy of M35 genes may confer adaptive advantages, enabling enhanced manipulation of host defenses and successful infection. Previous studies have shown that fungal M35s can interact with the plant immune system by inducing cell death [5] and inhibiting host chitinases [7, 8]. Consistent with these observations, our prior work revealed high gene abundance in other pathogenicity-associated families in Botryosphaeriales species, including the Necrosis- and Ethylene-inducing peptide 1-like protein (NLP) superfamily [13] and the pectin methylesterase (PME) gene family [19].

### Putative Effector M35s across Dothideomycetes

The widespread occurrence of putative effector M35 genes across Dothideomycetes species suggests their long-term retention in fungal genomes, likely due to their critical role in virulence. Functional studies have demonstrated that the M35 protein FocM35_1 from *Fusarium oxysporum* f. sp. *cubense* acts as an effector, inducing cell death and suppressing host chitinase activity [7]. Host chitinases degrade the chitin-rich fungal cell wall, releasing fragments that are recognized by plant receptors and trigger immune responses [9, 11]. The putative effector M35s identified here exhibited characteristic features, including an N-terminal signal peptide essential for secretion into the plant apoplast, as confirmed by molecular assays [7, 8].

A major distinction between putative non-effector and putative effector M35s is the absence of a classical signal peptide in the former. Our findings are consistent with earlier reports indicating that sequence modifications—such as indels—likely contribute to the emergence of putative non-effector M35s in Dothideomycetes gene families [13, 19]. Large indels in the 5’-region of putative non-effector M35 genes appear to disrupt the signal peptide sequence, preventing extracellular secretion.

### The Occurrence and Diversification of the M35 Gene Family in *Corynespora cassiicola*

The M35 gene family in *C. cassiicola* is relatively modest in size, with most isolates harboring up to three genes. This pattern is comparable to that in other polyphagous plant-pathogenic fungi, such as *V. dahliae* (two M35 genes) [5] and *R. cerealis* (four M35 genes) [8]. Given the established role of M35s as key virulence factors in various fungi [5–8], it is reasonable to hypothesize a similar contribution to pathogenicity in *C. cassiicola*.

The four sub-clades within the *C. cassiicola* M35 gene family (Fig. 2) exhibit distinct evolutionary trajectories shaped by biased gene retention and loss, a pattern observed in other Dothideomycetes gene families [13, 19]. Positive selection likely favored retention of functional paralogs with adaptive roles [39], whereas purifying selection eliminated nonfunctional paralogs carrying deleterious mutations [40]. Sub-clades Cc_M35_1.1 (Fig. 2b) and Cc_M35_2.1 (Fig. 2d) appear to have undergone biased retention, preserving members across most *C. cassiicola* isolates. In contrast, biased gene loss has reduced the prevalence of genes in sub-clades Cc_M35_1.2 (Fig. 2c) and Cc_M35_2.2 (Fig. 2e).

### The M35 Gene Family in the Context of the Effector Repertoire of *Corynespora cassiicola*

In addition to its broad host range, *C. cassiicola* possesses a substantial and diverse putative effector repertoire, which likely underpins its adaptability and virulence as a polyphagous pathogen [37, 41]. Our results highlight the inclusion of M35 family members within this repertoire. Despite sharing a conserved Peptidase_M35 domain, putative effector M35s in *C. cassiicola* have diversified considerably, accumulating substantial genetic variation over evolutionary time (Table 1). Effector genes typically evolve rapidly under strong selective pressure to evade host recognition and optimize function [42], a pattern consistent with other Dothideomycetes plant pathogens such as *Leptosphaeria maculans* [43] and *Zymoseptoria tritici* [44].

Effectors often contribute to host specialization by acquiring adaptations for specific host interactions [45]. Prior evidence suggests potential host specialization among *C. cassiicola* isolates [46, 47], possibly linked to effectors recognized by limited compatible hosts [41]. Notably, putative effector M35s in sub-clade Cc_M35_2.2 (Fig. 2e) were exclusively recovered from isolates associated with cotton plants. These proteins retain essential functional residues (Fig. 4b) beyond the Peptidase_M35 domain, suggesting retained activity and a possible specialized role in *C. cassiicola*–cotton interactions.

Putative non-effector M35s were comparatively rare in *C. cassiicola* and predominantly clustered within second-order sub-clades of Cc_M35_2.1 (Fig. 2d), indicating a shared evolutionary origin. Despite this common ancestry, these genes were detected in isolates from diverse hosts across multiple continents, potentially reflecting agriculturally mediated dispersal through global movement of infected plant material.

### The Expression Dynamics of M35 Genes during Soybean Infection by *Corynespora cassiicola*

Upregulation of M35 genes during infection is well documented in plant-pathogenic fungi [7, 8]. Our RT-qPCR results confirmed expression of all three M35 genes in *C. cassiicola* during soybean infection, supporting their potential involvement in pathogenicity. Although expression levels varied across the infection time course (0–8 dpi), a consistent trend emerged: peak expression occurred at 8 dpi, corresponding to late infection stages. This pattern differs from that reported for *F. oxysporum* f. sp. *cubense* [7] and *R. cerealis* [8], where M35 genes peaked early (0–2 dpi), highlighting species-specific expression dynamics.

Notably, CC_29_g1451 (sub-clade Cc_M35_2.1), classified as a putative non-effector due to the lack of a classical signal peptide, showed a small but statistically significant increase in expression at 8 dpi (Supplementary Figure S3). This suggests a possible intracellular role within the fungal cell that may contribute to infection. Overall, the upregulation of all three M35 genes during soybean infection underscores their likely functional importance in the pathogenic process of *C. cassiicola*.

## Conclusion

This study advances our understanding of the evolutionary history and functional roles of M35 metalloproteases in fungal pathogenicity. Most Dothideomycetes species possess a relatively small number of M35 genes, whereas Botryosphaeriales species exhibit a markedly larger repertoire, likely resulting from gene duplications. In *Corynespora cassiicola*, the M35 metalloprotease family displays considerable diversity, comprising four distinct sub-clades. Within *C. cassiicola*, sub-clades Cc_M35_1.1 and Cc_M35_2.1 show evidence of biased gene retention, with members present in the majority of isolates, while sub-clades Cc_M35_1.2 and Cc_M35_2.2 have undergone episodes of gene loss. The M35 genes of *C. cassiicola* exhibit distinct expression patterns during soybean infection, with the putative effector gene CC_29_g9669 showing the highest expression levels. These findings underscore the potential contributions of putative M35 effectors to the pathogenicity of *C. cassiicola*. However, further functional studies are needed to confirm their specific roles in fungal virulence. Overall, this work provides a foundation for future investigations into the mechanisms by which M35 effectors suppress host immune responses. 

## Supplementary Information

Below is the link to the electronic supplementary material.

Supplementary Material 1

Supplementary Material 2

Supplementary Material 3

## Data Availability

This study analyzed genomic data that is publicly available at https://mycocosm.jgi.doe.gov/mycocosm/home and https://www.ncbi.nlm.nih.gov/genbank/. Additional information that supports the findings of this study can be found in the supplementary material.
