# Supplementary material for: Evolutionary History and Diversification of M35 Metalloproteases in Dothideomycetes: A Phylogenomic Overview and Case Study in Corynespora cassiicola
Source: Curr Microbiol. 2026 Feb 21;83(4):204. doi: 10.1007/s00284-026-04772-x (PMC12924839; doi:10.1007/s00284-026-04772-x)
Supplement: Supplementary file 1 — Supplementary Material 1 [file 284_2026_4772_MOESM1_ESM.pdf]

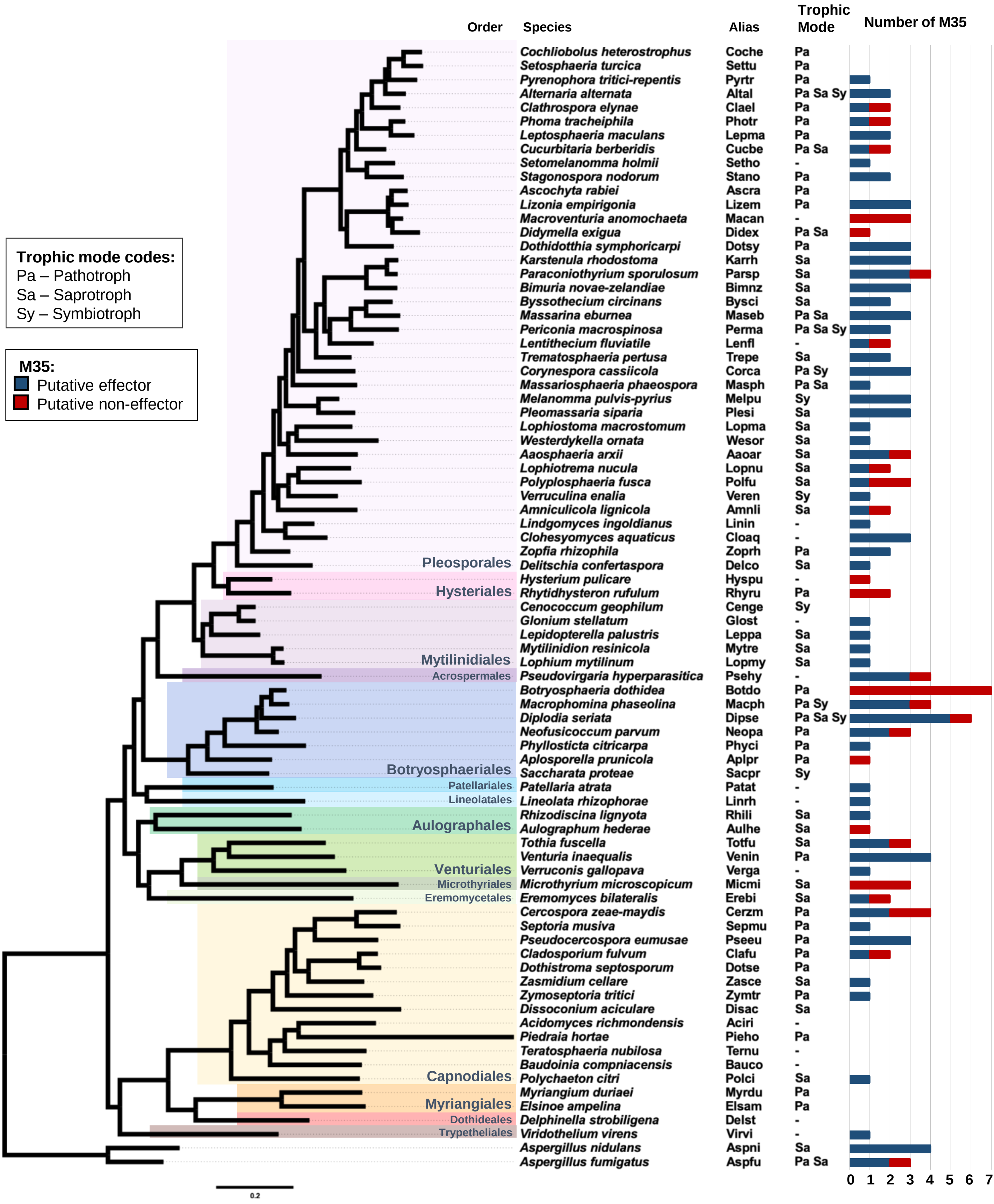

Fig. S1 Distribution of putative effector M35s and putative non-effector M35s among 79 species across the Dothideomycetes class of fungi. The Maximum-likelihood consensus tree was based on a dataset of 1851 single-copy ortholog proteins. Phylogenomic tree, Alias, and Trophic Modes are from a previous study (Dal Sasso et al. 2023)

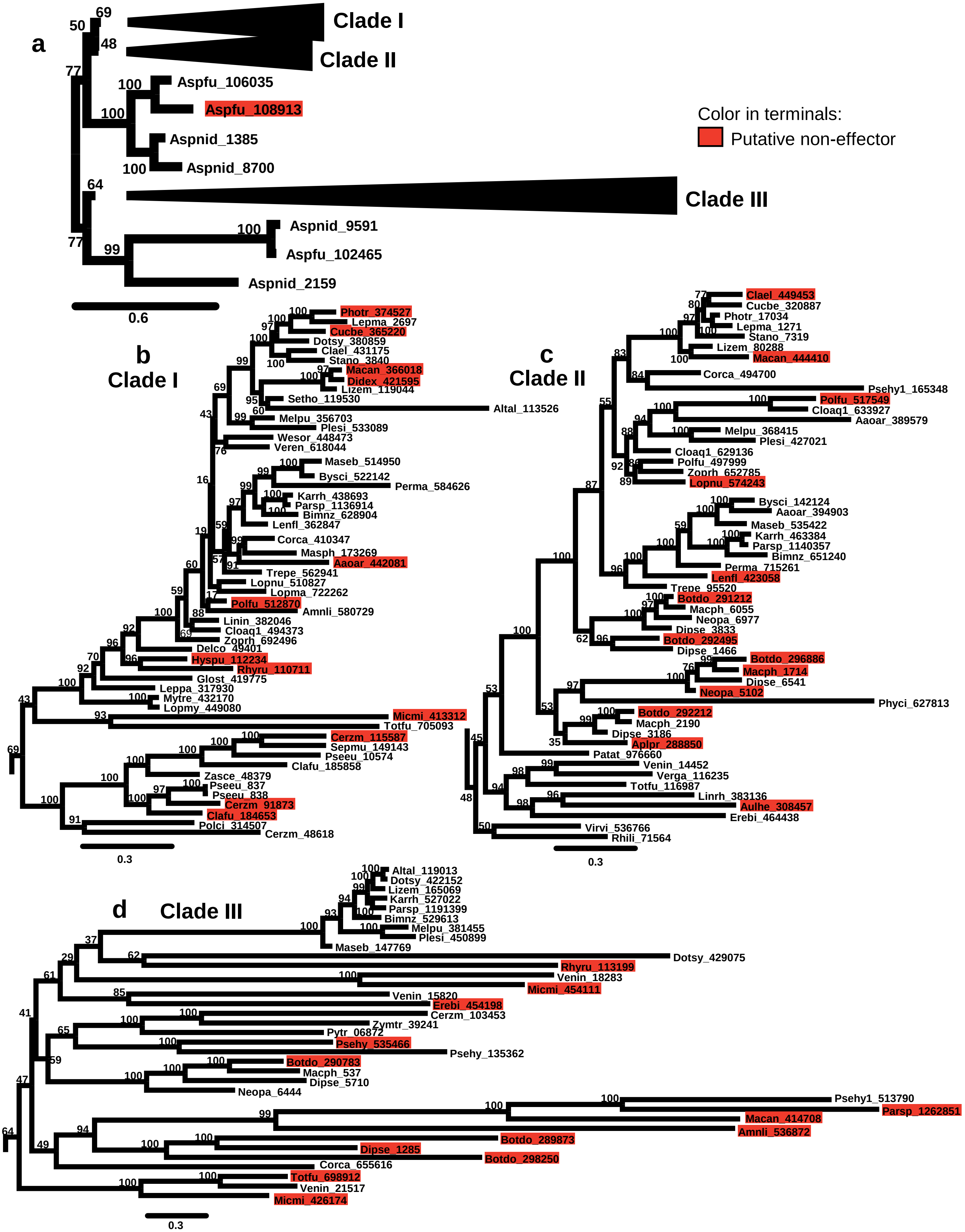

Fig. S2 Maximum likelihood tree of the Deuterolysin Metalloprotease (M35) gene family across the Dothideomycetes. The analysis was based on the full set of 146 protein sequences of M35s obtained from 65 species across the Dothideomycetes class of fungi, with *Aspergillus nidulans* and *A. fumigatus* as outgroups. **a** Overview of the unrooted consensus showing three major clades (collapsed into triangles) with low bootstrap values (<70). **b-d**. Expanded views show the internal composition of each clade. **b** Clade I. **c** Clade II. **d** Clade III. Nodal support values are given as bootstrap values above the branches. Branch lengths are drawn to scale. Scale bar corresponds to the expected number of substitutions per site. Red terminals indicate putative non-effector M35s

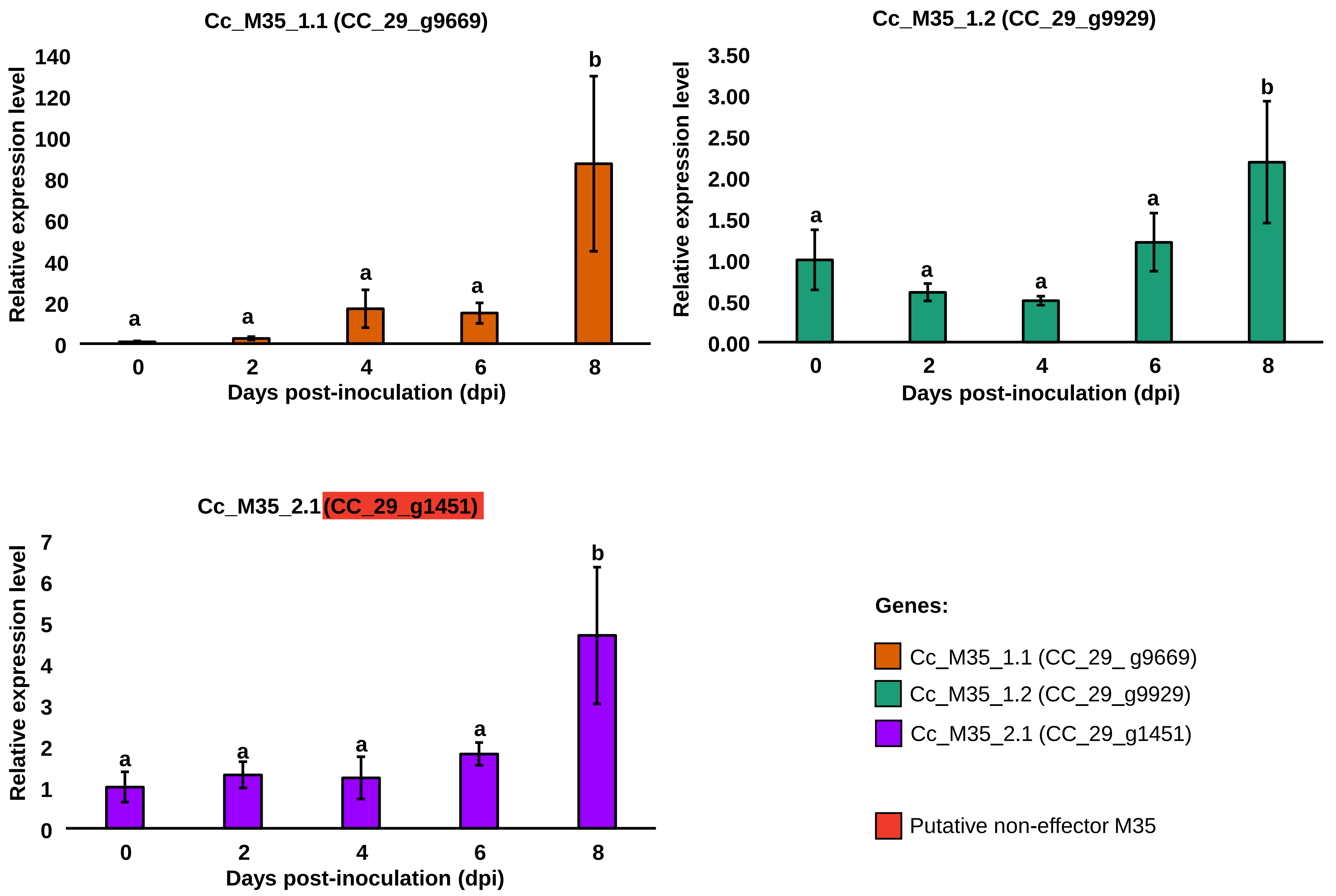

Fig. S3 Relative expression levels of three Deuterolysin Metalloprotease (M35) genes of *Corynespora cassiicola* isolate CC_29 on soybean leaves, during 0, 2, 4, 6, and 8 days post-inoculation (dpi), respectively. Data were obtained from the RT-qPCR analyses. The relative expression levels were calculated using the $2^{-\Delta\Delta Ct}$ method, with four biological replicates for each time point. The constitutive β-tubulin gene of *C. cassiicola* was used as an endogenous control. The relative expression level was calibrated and set as 1 at 0 dpi. The letters above bars indicate statistically significant differences according to Tukey's test (p-value < 0.05). Error bars show the standard error of the mean. Levels of expression were color-coded according to the three M35 genes, as indicated
