# Supplementary material for: Evolutionary History and Diversification of M35 Metalloproteases in Dothideomycetes: A Phylogenomic Overview and Case Study in Corynespora cassiicola
Source: Curr Microbiol. 2026 Feb 21;83(4):204. doi: 10.1007/s00284-026-04772-x (PMC12924839; doi:10.1007/s00284-026-04772-x)
Supplement: Supplementary file 3 — Supplementary Material 3 [file 284_2026_4772_MOESM3_ESM.pdf]

Table S3 Description of primer sequences used for RT-qPCR analysis of Deuterolysin Metalloprotease (M35) genes in *Corynespora cassiicola* (isolate CC_29).

**Gene name**	**Gene ID (CC_29)**	**Sequences 5’- 3’**	**References**
Cc_M35_1.1	CC_29_g9669	F: ATCTAGCACTAGCAGCACGG<br>R: TTGCAGTTTGTCGAGGTGGT	This study
Cc_M35_1.2	CC_29_g9929	F: CCCCTGACTCCCCTGATGTA<br>R: CAGCGAGGGGAAGTGGTATC	This study
Cc_M35_2.1	CC_29_g1451	F: ATTCTTCCTCCGGCATCGAC<br>R: CGGAAGTTCGACTGGAGGAC	This study
β-tubulin	CC_29_g4872	F: CAGACCGGTCAATGCGGTAA<br>R: GCCATTGTAGACGCCGGATC	Dal’Sasso et al. 2022

Table S4 Amino acid sequence alignment of two conserved segments for 146 Deuterolysin Metalloprotease (M35) proteins across Dothideomycetes. The alignment was generated by MAFFT v7.453. Arrows indicate amino acid positions, using the protein Aspnid_1387 of *Aspergilus nidulans* as a reference sequence. Code-color: yellow, zinc-binding amino acid residues; blue, catalytic amino acid residues. Protein ID with highlight in red: a putative non-effector M35.

	Segments	
**Protein ID**	**I**	**II**
	304 ↑	316 ↑
Aspnid_1385	HEFTH	GTDDLG-YG
Aspnid_8700	HEFTH	GTDDLG-YG
Aspfu_106035	HEFTH	GTDDYA-YG
Aspfu_108913	HEFTH	GTEDLG-YG
Bysci_142124	HEATH	GTDDHG-YG
Aaoar_394903	HEVTH	GTDDLG-YG
Maseb_535422	HEVTH	GTDDLG-YG
Perma_715261	HESTH	GTDDLG-YG
Karrh_463384	HEVTH	GTRDLG-YG
Parsp_1140357	HETTH	GTDDLG-YG
Bimnz_651240	HEVTH	GTGDLG-CG
Trepe_95520	HEVTH	GTQDYG-YG
Melpu_368415	HEMTH	GTDDYGTYG
Plesi_427021	HEMTH	GTEDYSTYG
Lopnu_574243	HETTH	GTSDYGVYG
Polfu_497999	HEMTH	GTSDYGVYG
Zoprh_652785	HETTH	GTSDYGAYG
Cloaq_629136	HEVTH	GTSDYGVYG
Lenfl_423058	HETMH	GADDLG-YG
Corca_494700	HEVTH	GTSDYGGYG
Clael_449453	HEMTH	GTSDYGGYG
Photr_17034	HEMTH	GTSDYGGYG
Lepmu_1271	HEMTH	GTSDYGGYG
Cucbe_320887	HETTH	GTSDYGGYG
Lizem_80288	HEMTH	GTSDYGGYG
Stano_7319	HEMTH	GTSDYGGYG
Macan_444410	HEMTH	GTSDYGGYG
Botdo_292495	HEMTH	GTTDQGAYG
Dipse_1466	HEMTH	GTTDQGAYG
Botdo_292212	HESTH	GTDDLG-YG
Macph1_2190	HESTH	GTDDLG-YG
Dipse_3186	HEATH	GTDDLG-YG
Aplpr_288850	HEFTH	GTDDLG-YG
Botdo_291212	HETTH	GTSDYGGYG
Macph_6055	HEVTH	GTSDYGGYG
Dipse_3833	HEMTH	GTSDYNGYG
Neopa_6977	HEMTH	GTDDFGGYG
Patat_976660	HEYTH	GTDDYA-YG
Virvi_536766	HEFTH	GTQDLG-YG
Rhili_71564	HEMTH	GTLDLG-YG
Totfu_116987	HEFTH	GTEDYA-YG
Venin_14452	HEYTH	GTEDNG-YG
Verga_116235	HEFTH	GTEDNG-YG

Continued Table S4

Botdo_296886	HEFTH	GTDDLG-YG
Dipse_6541	HEFTH	GTDDLG-YG
Neopa_5102	HEFTH	GTDDLG-YG
Macph_1714	HEFTH	GTDDLG-YG
Bysci_522142	HEETH	GTDDLG-YG
Maseb_514950	HEETH	GTDDLG-YG
Corca_410347	HEETH	GTDDYA-YG
Karrh_438693	HEETH	GTDDLG-YG
Parsp_1136914	HEETH	GTDDLG-YG
Bimnz_628904	HEETH	GTDDLG-YG
Lenfl_362847	HEETH	GTDDLG-YG
Trepe_562941	HEETH	GTDDIA-YG
Lopnu_510827	HEETH	GTDDLG-YG
Polfu_512870	HEETH	GTDDLG-YG
Linin_382046	HEETH	GTDDLG-YG
Cloaq_494373	HEETH	GTDDLG-YG
Lopma_722262	HEETH	GTDDIA-YG
Masph_173269	HEETH	GTDDFA-YG
Melpu_356703	HEETH	GTQDLG-YG
Wesor_448473	HEETH	GTDDLG-YG
Veren_618044	HEETH	GTDDLA-YG
Clael_431175	HEETH	GTQDLG-YG
Stano_3840	HEETH	GTQDNG-YG
Photr_374527	HEEAH	GTDDLG-YG
Cucbe_365220	HEEAH	GTDDLG-YG
Dotsy_380859	HEETH	GTDDLG-YG
Lepmu_2697	HEETH	GTDDLG-YG
Setho_119530	HEETH	GTEDLG-YG
Lizem_119044	HEETH	GTDDLG-YG
Macan_366018	HEETH	GTDDLG-YG
Didex_421595	HEETH	GTDDLG-YG
Zoprh_692496	HEETH	GTDDLG-YG
Plesi_533089	HEETH	GTEDNG-YG
Amnli_580729	HEEAH	GTDDLG-YG
Delco_49401	HEETH	GTDDLA-YG
Aaoar_442081	HEETH	GTDDLA-YG
Hyspu_112234	HENTH	GTQDYA-YG
Rhyru_110711	HETTH	GTDDYA-YG
Glost_419775	HENTH	GTQDYA-YG
Leppa_317930	HENTH	GTEDYG-YG
Mytre_432170	HESTH	GTDDNA-YG
Lopmy_449080	HESTH	GTDDNA-YG
Perma_584626	HEMTH	GTDDLG-YG
Cerzm_91873	HEETH	GTDDLG-YG
Pseeu_837	HEETH	GTQDYG-YG
Pseeu_838	HEETH	GTQDYG-YG
Clafu_184653	HEETH	GTQDYG-YG
Zasce_48379	HEETH	GTDDYA-YG
Clafu_185858	HEETH	GTEDFA-YG
Cerzm_115587	HEKTH	GTDDYA-YG
Sepmu_149143	HEKTH	GTDDYA-YG
Pseeu_10574	HEETH	GTEDYA-YG
Polci_314507	HEETH	GTEDNG-YG
Cerzm_48618	HETTH	NTVDYA-YG
Altal_113526	HETTH	STQDLA-YG

Continued Table S4

Linrh_383136	HEMTH	GTDDNA-YG
Polfu_517549	HEMTH	GTDDYGTYG
Cloaq_633927	HEMTH	GTDDYGTYE
Aulhe_308457	HEFAH	GTQDLG-YG
Erebi_464438	HEFTH	GTNDNG-YG
Aaoar_389579	HEFTH	GTDDYGVYG
Psehy_165348	HESTH	GTDDYG-YG
Phyci_627813	HEMTH	GTIDIG-YG
Micmi_413312	HEYTH	GTQDLA-YG
Totfu_705093	HEMTH	ETDDKT-MG
Totfu_698912	HEMTH	STDDFGHYG
Venin_21517	HEMTH	ATDDFGHYG
Micmi_426174	HETTH	STVDFNNYG
Corca_655616	HELTH	ATTDFA-YG
Botdo_290783	HELSH	PARDYA-IG
Macph_537	HELSH	RCQDFA-VG
Dipse_5710	HELAH	PARDYA-YG
Neopa_6444	HELTH	ATDDFA-AE
Aspnid_2159	HEYAH	GTEDIA-YG
Aspnid_9591	HELAH	ET-----YG
Aspfu_102465	HELAH	ET-----YG
Psehy_535466	HEMSH	ATGDYA-YG
Psehy_135362	HEFAH	WIVDVA-YF
Pytr_06872	HEMTH	ICSDYA-YG
Cerzm_103453	HELTH	TCDDLA-YG
Zymtr_39241	HEMAH	GCADYA-YF
Altal_119013	HEMTH	GTNDYG-YG
Dotsy_422152	HEMTH	GTDDYG-YG
Lizem_165069	HEMTH	GTEDYG-YG
Karrh_527022	HEFTH	GTTDYA-YG
Parsp_1191399	HEMTH	GTTDYA-YG
Bimnz_529613	HEMTH	GTGDYG-YG
Maseb_147769	HEMTH	GTDDWG-YG
Melpu_381455	HEFTH	GTDDWA-YG
Plesi_450899	HEMTH	GTTDWA-YG
Botdo_289873	HEVSH	ETRDWG-FL
Dipse_1285	HEMTH	ATDDIRHDK
Venin_15820	HELTH	STDDLA-YD
Botdo_298250	HEFLH	AARDYGHLW
Erebi_454198	HELMH	GLMDYA-YT
Venin_18283	HELTH	VTTDIT-YE
Micmi_454111	HELTH	PTVDHC-QG
Dotsy_429075	HEFSH	RLLDYA-YG
Rhyru_113199	HEYSH	AKQDWA-YG
Macan_414708	HEMHH	--TDYA-YG
Psehy_513790	HELTH	DDEDNP-YG
Parsp_1262851	HELTH	-TDDHT-YN
Amnli_536872	HELFH	DDADRC-YG
